# Termite-engineered microbial communities of termite nest structures: a new dimension to the extended phenotype

**DOI:** 10.1093/femsre/fuac034

**Published:** 2022-07-05

**Authors:** Hongjie Li, Chris Greening

**Affiliations:** State Key Laboratory for Managing Biotic and Chemical Threats to the Quality and Safety of Agro-products, Key Laboratory of Biotechnology in Plant Protection of Ministry of Agriculture and Zhejiang Province, Institute of Plant Virology, Ningbo University, Ningbo 315211, China; Department of Microbiology, Biomedicine Discovery Institute, Monash University, Clayton, VIC 3800, Australia; Centre to Impact AMR, Monash University, Clayton, VIC 3800, Australia; SAEF: Securing Antarctica’s Environmental Future, Monash University, Clayton, VIC 3800, Australia

**Keywords:** animal–microbe interactions, symbiosis, methane, Actinobacteria, ecosystem engineering

## Abstract

Termites are a prototypical example of the ‘extended phenotype’ given their ability to shape their environments by constructing complex nesting structures and cultivating fungus gardens. Such engineered structures provide termites with stable, protected habitats, and nutritious food sources, respectively. Recent studies have suggested that these termite-engineered structures harbour Actinobacteria-dominated microbial communities. In this review, we describe the composition, activities, and consequences of microbial communities associated with termite mounds, other nests, and fungus gardens. Culture-dependent and culture-independent studies indicate that these structures each harbour specialized microbial communities distinct from those in termite guts and surrounding soils. Termites select microbial communities in these structures through various means: opportunistic recruitment from surrounding soils; controlling physicochemical properties of nesting structures; excreting hydrogen, methane, and other gases as bacterial energy sources; and pretreating lignocellulose to facilitate fungal cultivation in gardens. These engineered communities potentially benefit termites by producing antimicrobial compounds, facilitating lignocellulose digestion, and enhancing energetic efficiency of the termite ‘metaorganism’. Moreover, mound-associated communities have been shown to be globally significant in controlling emissions of methane and enhancing agricultural fertility. Altogether, these considerations suggest that the microbiomes selected by some animals extend much beyond their bodies, providing a new dimension to the ‘extended phenotype’.

## Teaser

This review discusses how and why termites select for beneficial microbial communities by constructing mounds, nests, and fungal gardens, and explores the ecological and biogeochemical impacts of these termite-engineered communities.

## Introduction

**Figure 1.**
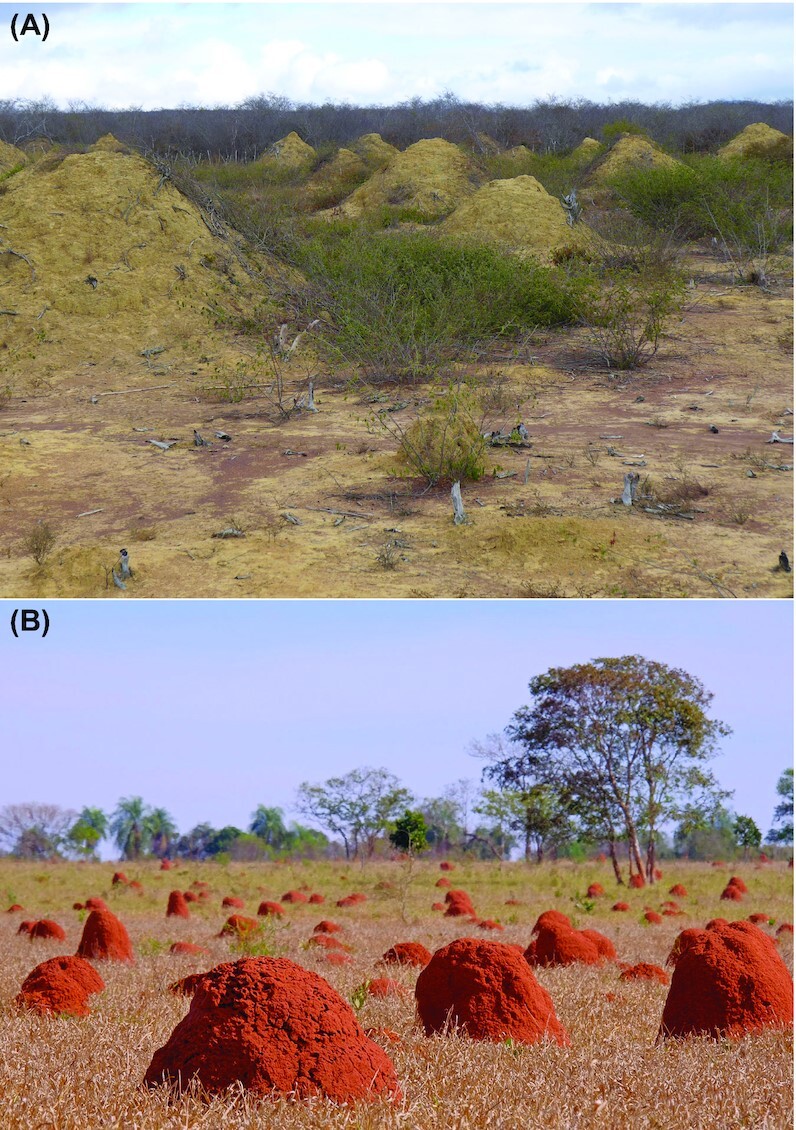
Photographs demonstrating the importance of termite mounds in tropical regions. (A) As ecologically dominant eusocial organisms, millions of enormous soil mounds constructed by *Syntermes* termites span 230 000 km^2^ of Northeast Brazil and persist for up to 4000 years (Martin et al. 2018). (B) Extensive termite mounds in a dry grassy field in Brazil.

Termites are a clade of eusocial cockroaches (order Blattodea; formerly Isoptera) with approximately 3000 described species (Krishna et al. 2013, Šobotník and Dahlsjö 2017, Evangelista et al. 2019). Among the most successful invertebrates, they span two-thirds of the Earth’s surface, dominate insect communities in the tropics, and at least rival humans in their global biomass (Jones and Eggleton 2011, Bar-On et al. 2018, Chouvenc et al. 2021). Termite colonies inhabit enclosed nests, which are either subterranean (underground nests), arboreal (tree-associated nests), or epigeal (soil mounds), depending on their lineage (Noirot and Darlington 2000, Šobotník and Dahlsjö 2017). Their diet comprises living or decaying plant materials from a range of sources, including wood, grasses, leaf litter, soil humus, and the faeces of herbivorous mammals (Donovan et al. 2001). All termites feed on plant-derived lignocellulose, a highly abundant but recalcitrant mixture of cellulose, hemicellulose, and lignin. Termites rely on mutualistic gastrointestinal microbiota to mediate lignocellulose hydrolysis and fermentation, resulting in production of acetate, hydrogen (H_2_), and methane (Breznak 1975, Odelson and Breznak 1983, Brauman et al. 1992, Watanabe et al. 1998, Leadbetter et al. 1999, Pester and Brune 2007, Brune 2014, Liu et al. 2019). Termites thus function as ‘metaorganisms’ dependent on host–microbe symbioses (Bosch and McFall-Ngai 2011). In combination, the capacity of termites to build complex nests, develop eusocial systems, efficiently degrade lignocellulose, and maintain symbiotic microbiota underpins their global prosperity. While some termite species are major pests, most are important soil ecosystem engineers that mediate key ecosystem services, including by enhancing carbon and nutrient cycling, modifying soil structures, and increasing dryland fertility and resilience (Dangerfield et al. 1998, Abe et al. 2000, Jouquet et al. 2011). In turn, the broad environmental influence of termites and their microbial symbionts provides a dramatic example of the ‘extended phenotype’ (Fig. 1; Dawkins 2016).

Termite evolution is proposed to have been driven by shifts in microbial symbionts and nesting structures. In the defining event of termite evolution, eusocial wood-feeding termites are thought to have diverged from their cockroach ancestors by acquiring gut cellulolytic flagellate protists approximately 150 million years ago (Lo et al. 2000, Inward et al. 2007, Engel et al. 2010, Krishna et al. 2013, Bucek et al. 2019). This evolutionary innovation facilitated wood digestion, nest development, eusocial organization, and population expansions (Grimaldi and Engel 2007, Aanen and Eggleton 2017, Chouvenc et al. 2021). The deeper-branching families of termites (‘lower’ termites) are subterranean or arboreal lineages possessing protist symbionts (Bucek et al. 2019). In contrast, the diverse ‘higher’ termites that evolved approximately 50 million years ago (clade Termitidae) are mound-building species that lack protist symbionts. Comprising approximately 70% of all described termite species, the Termitidae adopt a wide range of diets, with soil-feeding, litter-feeding, wood-feeding, and fungus-farming species described. It has been argued that, through losing their obligate gut protist symbionts, the Termitidae were free to diversify their diets, lifestyles, and gastrointestinal anatomies (Krishna et al. 2013, Brune 2014, Aanen and Eggleton 2017, Šobotník and Dahlsjö 2017, Bucek et al. 2019). The soil-, litter-, and wood-feeding lineages rely on gastrointestinal bacterial symbionts to digest soil organic matters and plant materials, respectively (Warnecke et al. 2007, Brune 2014, Moreira et al. 2018). In addition, the fungus-farming termites (Macrotermitinae) uniquely cultivate symbiotic fungi *Termitomyces* on their fungal gardens as food within their mounds and form an external (extracorporeal) digestive system (Wood and Thomas 1989, Rouland-Lefèvre and Bignell 2001). Thus, by forming new microbial mutualistic associations, adapting their anatomies, and expanding their dietary requirements, termites in turn were able to occupy new niches and increase their ecological breadth (Aanen and Eggleton 2017, Šobotník and Dahlsjö 2017).

While the significance of termite-associated symbiotic microbial communities and nesting structures is well-established, the critical role of microorganisms within nesting structures is only starting to be recognized. A combination of culture-based and culture-independent studies have revealed specialized bacterial communities are selected within termite mounds, nests, and fungal gardens (Fall et al. 2007, Otani et al. 2016, Enagbonma et al. 2019, Chen et al. 2020, Chiri et al. 2021, Soukup et al. 2021). Emerging evidence suggests these bacteria are selected by termites through various processes, contribute to the resilience of termite colonies, and influence wider ecological processes such as greenhouse gas cycling through their metabolic activities (Khalil et al. 1990, Holt 1998, Mathew et al. 2012, Visser et al. 2012, Poulsen et al. 2014, Nauer et al.2018b, Enagbonma and Babalola 2019a, Murphy et al. 2021, Schmidt et al. 2022, Witasari et al. 2022). These findings provide new insights into the evolutionary adaptations and ecological significance of termites. More broadly, they highlight a new concept in the evolutionary ecology of microbiomes: selection of beneficial environmental microbial communities provides a novel mechanism for animals to control their surroundings and further extend their phenotypes (Dawkins 2016, Dunn et al. 2020). This review synthesizes current knowledge on the selection, composition, roles, and significance of microbial communities in termite-engineered structures.

## Structure, formation, and role of termite nesting structures

In contrast to their subsocial wood-feeding cockroach sister lineages, termites construct nesting structures that are critical for many aspects of their lifestyles. These nests are highly diverse in size and architecture, spanning relatively simple arboreal and subterranean nests to complex cathedral mounds that tower metres high, with this diversity reflecting variations in the social organization, colony size, and feeding habits across termite species (Korb 2010). They also vary in their material composition, from the paper-like nests produced by Nasutitermitinae to the cement-like mounds formed by the Termitidae and some Rhinotermidae. In all termite species, except the earliest branching species *Mastotermes darwiniensis*, these nesting structures facilitate a caste system with an elaborate division of labour, with reproductive queens and kings, sterile workers, and in certain species specialized soldiers (Nalepa 1994, 2015). Nesting structures also provide defences against predators and buffering to environmental change, with the elaborate structures of termite mounds conferring stable microclimates in which temperature, humidity, and ventilation are well-managed (Lüscher 1961, Singh et al. 2019). Termite mounds of certain species also typically contain chambers to store foods and, for fungus-farming termites, cultivate fungi for food (Holt and Lepage 2000, Schmidt et al. 2014).

**Figure 2.**
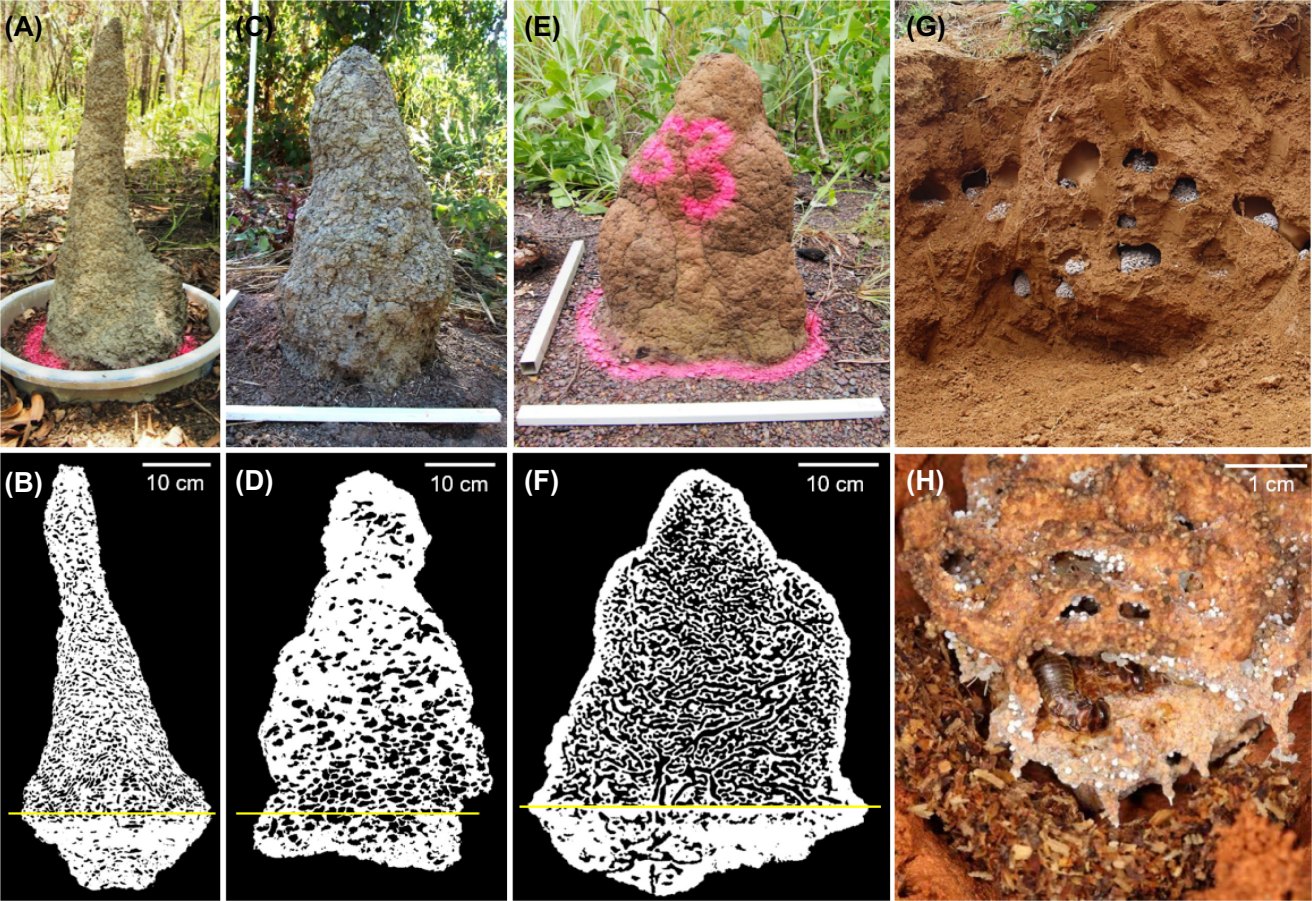
Photographs and tomographs of the complex internal structures and external morphologies of nesting structures across termite species. The external and internal structures of three north Australian termite species are shown, namely the wood-feeding *Microcerotermes nervosus* (A) and (B), soil-interface feeding *Macrognathotermes sunteri* (C) and (D), and grass-feeding *Tumulitermes pastinator* (E) and (F) are shown. The cross-section binary images are based on X-ray computer tomography (CT scanning; Nauer et al. 2018a). Also shown are the subterranean nests (G) and fungal garden (H) of the fungus-farming species *Odontermes formosanus* (Li et al. 2017).

Termite mounds are formed from pellets of soil, wood, and/or faecal material that are transported by colony workers and then compacted together using saliva. The external walls (mound periphery) typically form a solid but porous cement with a high proportion of inorganic matter, whereas the internal nesting structures (mound core) are built primarily from faecal material or soil depending on the species. Mounds contain complex networks of internal chambers that facilitate gas exchange, harbour the colony, and allow food storage or fungiculture (Gillman et al. 1972, Bruinsma 1979, Wood 1988, Korb 2010). Bacterial communities are also active within the peripheries, cores, and undersides of mounds, where they perform a range of functions (Chen et al. 2020, Chiri et al. 2021). To illustrate the complex and variable structures of termite mounds, Fig. 2 compares the internal and external mound structures of three representative Australian termite species (Nauer et al. 2018a, 2018b).

Macrotermitinae species also cultivate *Termitomyces* fungi in so-called fungus gardens within their mounds. To do so, these termites construct complex combs from their faeces within chambers either within or adjacent to the hive. Figure 2 shows the structures of these combs within the nest of the termite *Odontotermes formosanus*. In addition to providing a physical structure, termite faeces also contain a mixture of fungal asexual spores that inoculate the combs and partially digested plant materials that promote *Termitomyces* growth (Leuthold et al. 1989). Altogether, termites, gut microbiota, and fungi form an obligate tripartite mutualism that enables almost complete lignocellulose digestion through subdivision of labour: the fungi benefit from an optimal microclimate and accessible substrate supplies, whereas the termite and their gut microbiota gains benefit from a dependable and nutritious food source (Poulsen et al. 2014, Li et al. 2017, Ahmad et al. 2021). Though *Termitomyces* monoculture is maintained in the presence of termites, combs become infected by fungal parasites such as *Pseudoxylaria* that inhibit *Termitomyces* growth in the absence of termites (Shinzato et al. 2005, Guedegbe et al. 2009, Visser et al. 2009, 2011, Mathew et al. 2012, Bos et al. 2020). It is hypothesized that the bacterial communities within the fungal combs contribute to the maintenance of symbiosis (Mathew et al. 2012, Otani et al. 2016, Benndorf et al. 2018). It should be noted that another termite species, *Sphaerotermes sphaerothorax*, as the sole representative of the Sphaerotermitinae (sister family to Macrotermitinae), instead cultivates bacterial combs as a food source (Garnier-Sillam et al. 1989, Bucek et al. 2019); however, detailed studies have yet to be performed on the composition and function of its combs.

## Microbial composition and roles in termite mounds and nests

**Figure 3.**
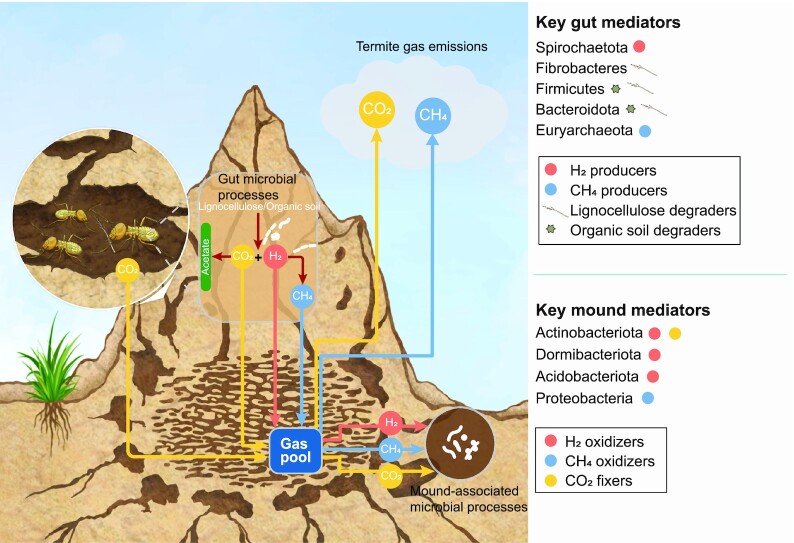
Overview of the microbial mediators of key biogeochemical processes in termite guts and mounds. The diagram shows how termite gut microbial processes results in the conversion of lignocellulose and other organic matter into the gases carbon dioxide (CO_2_), hydrogen (H_2_), and methane (CH_4_). Mound-associated bacteria consume these gases as energy and carbon sources, though there are still significant emissions of the greenhouse gases carbon dioxide and methane into the atmosphere. The key microbial phyla that mediate the consumption and production of these gases, as well as lignocellulose degradation, are also shown.

Termite mounds host highly adapted and active microbial communities (Fig. 3). Early studies on the African soil-feeding termite *Cubitermes niokoloensis* showed that the termite mounds were dominated by Actinobacteriota, whereas Firmicutes and Proteobacteria dominate the guts of termites and the surrounding soil, respectively (Fall et al. 2004, 2007). Similarly, Actinobacteriota-dominated communities have been observed through amplicon sequencing the mounds of various other soil-, wood-, and grass-feeding termites and the subterranean nests of the soil-feeder *Procornitermes araujoi* (Manjula et al. 2016, Moreira et al. 2018, Enagbonma et al. 2019, 2020, Chiri et al. 2021). Concordant findings have also been made through metagenomic analysis: Actinobacteriota and candidate phylum Dormibacterota comprise an average of 71% and 6.8% (57% and 7.5% based on amplicon sequencing) of bacteria in the mounds of the three dominant termite feeding groups of Australia (Chiri et al. 2021). Mounds also host various archaea (primarily Nitrososphaerales) and fungi (primarily Ascomycetota; Roose-Amsaleg et al. 2004, Costa et al. 2013, Wakung'oli et al. 2020, Chiri et al. 2021, Yan et al. 2021). These culture-independent insights are also supported by the isolation and characterization of various actinobacterial, acidobacterial, and proteobacterial strains from termite mounds and nests (Sujada et al. 2014, Sujarit et al. 2017, Lin et al. 2020, Oberpaul et al. 2020).

Where do termite mound communities originate from and how are they assembled? Most bacteria and fungi inhabiting termite mounds are also present in surrounding soil, suggesting they are recruited by termites from environmental sources during mound construction (Chen et al. 2020, 2021, Chiri et al. 2020, 2021). However, the communities are generally less rich and more even in mounds compared to soils (Makonde et al. 2015, Guimaraes et al. 2020, Chen et al. 2021, Chiri et al. 2021). This reduced alpha diversity is driven by expansions of certain mound-associated microorganisms, primarily though not exclusively from the Actinobacteriota, Dormibacterota, and Ascomycetota. Whereas most soil-associated rare taxa from multiple phyla in the termite mounds are substantially lost. As detailed below, this filtering is likely due to a combination of mound physicochemical features, termite metabolic activity, and increased interspecies competition, compounded by the relative spatial similarity and temporal stability of termite mounds compared to soil communities. Consistently, based on large-scale ecological analysis of 134 mounds in northern Australia, bacterial and fungal communities are primarily assembled through deterministic rather than neutral processes (Chen et al. 2020). Community composition is similar between mound cores and peripheries, even though bacterial abundance is lower in the cement-like material of mound periphery (Chiri et al. 2020, 2021).

**Figure 4.**
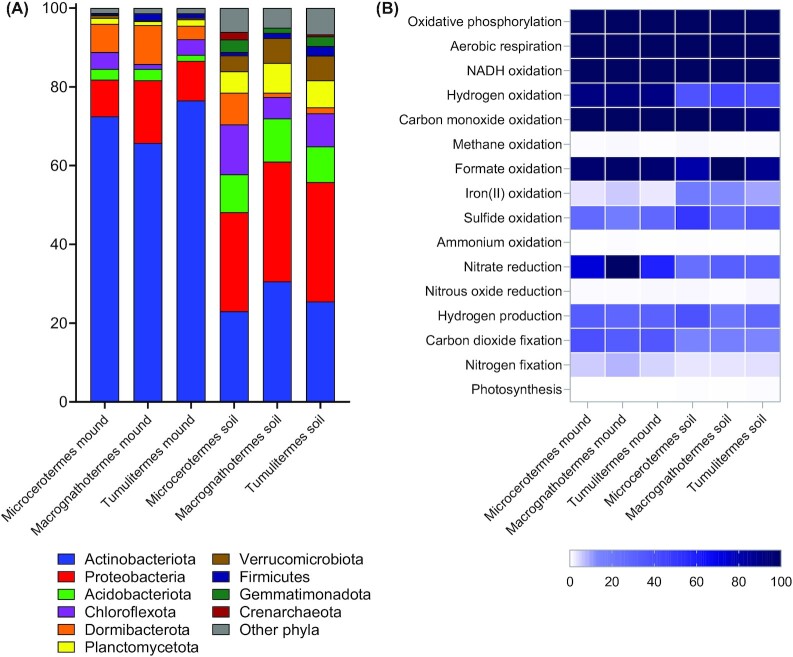
Composition and metabolic capabilities of the microbial communities of termite mounds and surrounding soils. (A) Relative abundance of microbial phyla based on read mapping of metagenomes using GraftM. (B) Percentage of microbial communities that mediate different metabolic processes. Results are shown for three different termite species, namely the wood-feeding *Microcerotermes nervosus*, soil-interface feeding *Macrognathotermes sunteri*, grass-feeding *Tumulitermes pastinator*, as previously described (Chiri et al. 2021).

Termite mound communities have distinct metabolic capabilities and activities compared to soils (Fig. 4). Similarly to soils, metagenomic and biogeochemical studies suggest most mound-associated bacteria use organic compounds as carbon and energy sources, in line with reports of high heterotrophic activity in mounds (Holt 1998, Ndiaye et al. 2004a, Chiri et al. 2021). However, a much higher proportion of mound compared to soil bacteria can grow using H_2_ as an energy and electron source (Bay et al. 2021, Chiri et al. 2021): 91% of mound communities encode [NiFe]-hydrogenases to use molecular H_2_ as a substrate for aerobic respiration, and over a third of these can also use H_2_ to fix carbon dioxide into biomass through the Calvin–Benson–Bassham cycle. Consistently, both *in situ* and *ex situ* activity measurements show that mound communities rapidly consume H_2_, such that they recycle all H_2_ emissions from termite gastrointestinal fermentation and even serve as net sinks for atmospheric H_2_ (Khalil et al. 1990, Chiri et al. 2021). Carbon monoxide may also be an important energy source for mound-associated bacteria (Khalil et al. 1990, Chiri et al. 2021). This high capacity for chemolithoautotrophy is evident from metagenomic analysis of both mounds of three distinct Australian termite species and subterranean nests of an African species (Enagbonma et al. 2020, Chiri et al. 2021). These results are supported by metabolic annotation of metagenome-assembled genomes, which indicates most mound-enriched Actinobacteriota, Dormibacterota, and Acidobacteriota bacteria can grow mixotrophically using organic and inorganic compounds (Chiri et al. 2021). Capacity for nitrate reduction is also 6-fold higher in mounds compared to soils, suggesting mound bacteria can adapt to variations in oxygen availability in mounds by switching between aerobic and anaerobic respiration (Chiri et al. 2021). A small proportion of bacteria within termite mounds also appear to mediate the biogeochemically important processes of nitrification, methane oxidation, and possibly nitrogen fixation (Ndiaye et al. 2004b, Chiri et al. 2020, 2021, Lin et al. 2020).

In turn, the productivity and efficiency of termite mounds is likely to be significantly enhanced by mound-associated communities. It is generally thought that mounds are more carbon-, nitrogen-, and phosphorus-rich than surrounding soils due to the use of faecal material to build mound walls (López-Hernández et al. 1989, López-Hernández 2001, Brossard et al. 2007, Enagbonma et al. 2019). However, mound-associated bacteria may contribute to the availability of these nutrients by mediating carbon fixation, nitrogen fixation, and phosphate solubilization (Lin et al. 2020, Chiri et al. 2021). The energetic efficiency of termites is likely further enhanced by the recycling of H_2_ and carbon dioxide, produced during termite gut fermentation, into degradable organic compounds by mound-associated hydrogenotrophs (Chiri et al. 2021). Efficiency might also be increased through the use of carbon monoxide, released through biological processes and abiotic oxidation of organic compounds, as an energy and carbon source (Khalil et al. 1990, Chiri et al. 2021). As elaborated below, the metabolic capabilities of mound-associated bacteria also underlie their capacity to regulate greenhouse gas emissions and potentially enhance soil fertility.

Termites potentially rely on nest-associated bacteria as defensive symbionts. Nest-associated antimicrobials provide an additional level of protection for termites against parasitic fungi and bacteria beyond their nest physical structures, gut symbionts, and social immunity. Various isolates from termite mounds and nests, primarily from the phylum Actinobacteriota, produce bioactives and exhibit antifungal or antibacterial properties (Sujada et al. 2014, Chauhan et al. 2016, Krishanti et al. 2018, Hussaini et al. 2021). For example, *Streptomyces* are ubiquitous in the subterranean nest of *Coptotermes formosanus* and, in an experimental study, enhanced survival of termites exposed to the pathogenic fungus *Metarhizium robertsii* (Chouvenc et al. 2013, 2018). Moreover, termite colonies reared in nonsterile soil have a much higher survival rate (80%) than in sterile soils (22%; Chouvenc et al. 2018). Metagenomic analysis also indicates that *Pseudonocardia* species from phylum Actinobacteriota (Currie et al. 1999, Li et al. 2018, Van Arnam et al. 2018), known to be major defensive symbionts in fungus-farming ants, are highly enriched in the mounds of multiple Australian termite species (Chiri et al. 2021). Mound-associated bacteria, therefore, potentially provide ‘external immunity’ for termites (Rozen 2014). However, there is currently insufficient evidence to determine whether antimicrobial production by mound-associated Actinobacteriota is relevant *in situ* or provides termites with a competitive advantage. More detailed studies are ultimately required to systematically investigate the genetics, chemistry, and ecology of antimicrobial production in this context.

## Microbial composition and roles in fungal gardens

Fungal gardens harbor microbial communities distinct from those of surrounding mounds (Otani et al. 2016, Liang et al. 2020, Yang et al. 2021). A large-scale amplicon sequencing survey compared the bacterial communities of 33 combs from four major fungus-farming termite species (Otani et al. 2016). Together with several comparative studies, this research suggests that whereas mound cores and peripheries are dominated by soil-derived bacteria, comb communities contain high levels of bacteria from both faecal and environmental sources, including Firmicutes, Bacteroidota, Proteobacteria, and Actinobacteriota (Makonde et al. 2015, Li et al. 2016, Otani et al. 2016, Liang et al. 2020). Actinobacteriota are nevertheless relatively enriched in combs compared to guts and are likely primarily of environmental origin (Otani et al. 2016, Murphy et al. 2021). Based on beta diversity ordinations, the gut and comb communities both clustered by termite species, though there is much greater intraspecific variation in comb compared to gut communities (Otani et al. 2016). Consistent with these culture-independent studies, *Streptomyces, Micromonospora*, and *Bacillus* strains have also been isolated from combs (Mathew et al. 2012, Visser et al. 2012, Murphy et al. 2021). Genomic analyses of comb-enriched actinobacterial isolates suggest a high capacity for both lignocellulose hydrolysis and antimicrobial production, though metagenomic studies would be required to gain a more holistic insight into the capabilities of these communities (Murphy et al. 2021). In stark contrast to the diverse bacteria communities in combs, *Termitomyces* species comprise over 99.9% of the total fungal community based on amplicon sequencing, with considerable variation in *Termitomyces* ITS sequence types present within and between colonies (Shinzato et al. 2005, Otani et al. 2019, Yang et al. 2021).

Fungal comb communities significantly contribute to lignocellulose degradation, which is consistent with the role of the fungus garden as an extracorporeal digestive system. A recent study used ^1^H–^13^C correlation nuclear magnetic resonance (NMR) to chemically analyze the degree of pretreatment of lignin and polysaccharides during the fungus garden maturation in *O. formosanus* laboratory colonies; this revealed that the fungus-comb microbiome contributes to significant polysaccharide and lignin cleavage, leaving the mature comb enriched in more digestible cellulosic oligomers that are eventually ingested by termite host (Li et al. 2017). Although the specific roles in lignocellulose pretreatment of bacteria and fungi remain unclear, lignocellulolytic enzymes have been identified in the fungus garden microbiome. The genomes of comb-enriched actinobacterial isolates encode numerous carbohydrate-active enzymes (CAZymes) targeting the cellulose, hemicellulose, and pectin components of lignocellulose (Murphy et al. 2021). Diverse enzymes of *Termitomyces* origin are also present in the fungal gardens (Poulsen et al. 2014, da Costa et al. 2018). Altogether, this suggests that the efficient lignocellulose degradation in fungus-farming termites depends on a subdivision of labour between the termite, fungus, gut bacteria, and comb bacteria through a multipartite symbiosis (Ahmad et al. 2021).

A further key role of termite comb communities is to maintain monoculture of the symbiotic fungus *Termitomyces* through antimicrobial production. It was originally proposed, through the ‘gut sanitation’ hypothesis, that termite gut transit maintains monoculture by promoting *Termitomyces* growth and inhibiting fungal antagonists (Poulsen 2015); however, this hypothesis is increasingly disfavoured given fungal antagonists survive gut passage and have a relatively short transit time (Bos et al. 2020, Murphy et al. 2021). Instead, comb communities in active mounds most likely primarily mediate this function. Consistently, comb extracts, actinobacterial isolates, *Bacillus* isolates, and *Termitomyces* fungi themselves each exhibit strong antifungal and/or antibacterial activities (Mathew et al. 2012, Visser et al. 2012, Benndorf et al. 2018, Otani et al. 2019, Witasari et al. 2022). To date, approximately 400 natural products and numerous biosynthetic gene clusters have been discovered through studies of the fungus-farming termite symbiosis; these include polyketides and lanthipeptides of actinobacterial origin, as well as terpenes and alkaloids isolated from *Termitomyces* (Benndorf et al. 2018, Murphy et al. 2021, Schmidt et al. 2022). These products are comprehensively discussed in a recent review (Schmidt et al. 2022). Despite these advances, we presently lack a detailed mechanistic understanding of how specific bacterial species influence fungal dynamics. There is ample precedent for specific actinobacterial mutualists controlling fungal symbioses in other insects, as a result of convergent evolution, most notably studies showing antibiotic-producing *Pseudonocardia* specifically inhibit parasitic fungi and, thereby favour the fungal cultivar growth in leaf-cutting ants (Currie et al. 1999, Scott et al. 2008, Kaltenpoth 2009, Sen et al. 2009). However, it appears that termites are likely more promiscuous in the defensive symbiont associations that they form (Chouvenc et al. 2018).

## Mechanisms of termite-mediated microbial engineering

Termites select the composition and activities of their microbial communities in multiple ways. Initially, termites recruit microbial communities to their nesting structures by either transporting them from environmental sources (e.g. soil, wood, and leaves) or, primarily in the case of fungus-farming termite combs and subterranean *Coptotermes* nests, depositing them *via* their faecal material (Du et al. 2016). The combination of physicochemical conditions, organic and inorganic nutrients, and antimicrobial properties provided by termite-inhabited nesting structures in turn enables the most adapted bacteria to thrive and leads to the exclusion of potential pathogens. Through this environmental filtering, termite mounds develop highly abundant but less diverse microbial mound communities than surrounding soils (Chen et al. 2020, 2021, Chiri et al. 2021). Termite-regulated environmental filtering also likely underlies *Termitomyces* monoculture in fungal combs (Shinzato et al. 2005).

Termites promiscuously recruit symbionts into their nesting structures. For example, a landmark paper demonstrated that the subterranean termite *C. formosanus* opportunistically recruits potentially defensive *Streptomyces* from surrounding soils and provides favourable nutrients for their proliferation (Chouvenc et al. 2013, 2018). Reflecting this finding, 49 of the 83 *Streptomyces* isolates from nest material shared identical 16S rRNA gene sequences with isolates from surrounding soils, with this proportion likely to increase with more exhaustive sampling. In support of horizontal transmission, the majority of *Streptomyces* were not acquired in a vertical transmission assay (Chouvenc et al. 2018). Given these findings, how do termites guarantee acquisition of potential defensive symbionts? As described by Chouvenc et al. (2018), this *Coptotermes*–*Streptomyces* association and potential mutualism is ‘ubiquitous yet opportunistic and dynamic’; the ubiquity and diversity of free-living soil streptomycetes ensures associations can persist wherever termites are available to recruit them (Perret et al. 2000, Moran and Sloan 2015, Chouvenc et al. 2018). Consistently, based on analysis of 3348 isolates from 20 nests from eight different locations, termite colonies contain on average 48 *Streptomyces* morphotypes; no morphotypes were shared between locations and multiple isolates exhibited broad-spectrum antifungal activity (Chouvenc et al. 2018). There is also evidence of opportunistic recruitment of other widespread soil bacteria, notably methanotrophs and hydrogenotrophs, into mounds (Chiri et al. 2020, 2021, Chen et al. 2021). In contrast, vertical transmission remains a key mechanism for maintenance of termite gut mutualists, as well as the deposition of *Termitomyces* spores and gut bacteria in fungal combs (Korb and Aanen 2003, Aanen et al. 2009, Abdul Rahman et al. 2015, Bourguignon et al. 2018).

The physicochemical properties of termite nesting structures have multifaceted influences on environmental filtering. Long considered as unique islands with distinct physicochemical properties from the surrounding soils, mounds typically have higher levels of organic carbon, nitrogen compounds, bioavailable phosphorus, cations, carbon dioxide, and reduced gases (Wood 1988, Khalil et al. 1990, Garnier-Sillam and Harry 1995, Harry et al. 2001, Fall et al. 2007, Yan et al. 2021). Termite nesting structures are also texturally distinct, with 4-fold higher levels of silt compared to surrounding soils (Harry et al. 2001). Furthermore, the structure of complex mounds modulates temperature, humidity, and ventilation, providing a microclimate distinct from exposed soils (Lüscher 1961, Singh et al. 2019). These physicochemical factors drive deterministic selection of terrestrial microbial communities worldwide, and will variably influence growth and survival dynamics of specific microorganisms in termite nesting structures (Fierer 2017). For example, mound microporosity is the strongest predictor of methanotroph abundance and activity (Nauer et al. 2018a, Chiri et al. 2020). It should also be noted that there is typically less spatial heterogeneity and temporal variation in the physicochemical conditions of mounds compared to soils, which may result in exclusion of microorganisms with narrow niches and intensify competition. For example, in fungus-farming termites, mound microclimates are also thought to differentially influence the growth dynamics of *Termitomyces* species compared to fungal parasites (Katariya et al. 2018, Vesala et al. 2019).

Termites also continually sustain growth of symbionts by excreting metabolic endproducts. They release substantial quantities of the diffusible gases H_2_, methane, ammonia, carbon dioxide, and potentially carbon monoxide during lignocellulose digestion due to activities of their gut microbiota (Rasmussen and Khalil 1983, Khalil et al. 1990, Williams et al. 1994, French et al. 1997, Ji and Brune 2006). These gases in turn stimulate the growth of mound-associated hydrogenotrophic, methanotrophic, and carboxydotrophic bacteria, as well as nitrifying archaea (Ho et al. 2013, Chiri et al. 2020, 2021). H_2_ exchange provides a particularly strong basis for the opportunistic selection of defensive actinobacterial symbionts. As the central intermediate in termite-mediated lignocellulose degradation, termites emit up to 1.5 µmol H_2_ per gram per hour (scaling to up to 200 teragrams globally per year), all of which is rapidly consumed to subatmospheric levels by mound communities (Fig. 3; Zimmerman et al. 1982, Khalil et al. 1990, Sugimoto et al. 1998a, Pester and Brune 2007, Chiri et al. 2021). Consistently, culture-based studies have shown aerobic respiration of H_2_ sustains the growth and survival of Actinobacteriota, including antimicrobial producers such as *Streptomyces* and *Pseudonocardia* known to be enriched in mounds (Constant et al. 2010, Grostern and Alvarez-Cohen 2013, Chiri et al. 2021, Greening et al. 2021). Moreover, even prior to the discovery that mound-associated bacteria are hydrogenotrophs, microcosm studies established elevated levels of H_2_ primarily stimulate actinobacterial growth residing in upland soils (Osborne et al. 2010, Xu et al. 2021). Termites appear to rely on this effect to sustain actinobacterial symbionts. Consistently, termite activity is an extremely strong predictor of the H_2_ oxidation rates of mound-associated bacteria, providing a mechanistic basis for a dynamic and promiscuous symbiosis (Chiri et al. 2021). The percentage levels of carbon dioxide that occur in termite mounds, due to both termite respiration and gastrointestinal fermentation, likely also favours chemolithoautotrophic growth of mound bacteria (Khalil et al. 1990, Sanderson 1996, Jamali et al. 2013, Chiri et al. 2021).

Organic carbon liberated by termites also drives the composition of the microbiota in fungal gardens and likely termite mounds. For fungal combs, the first microbiome inoculation is performed by the termite: younger workers ingest undigested lignocellulosic material together with fungal nodules that contain asexual spores, then excrete this mixture to build a fresh garden (Leuthold et al. 1989, Li et al. 2016, 2017, 2021). Fungus-farming termites further promote this mutualism through providing a constant supply of lignocellulose, i.e. digested through a subdivision of labour between termites, gut microbiota, comb microbiota, and *Termitomyces* (Poulsen et al. 2014, Li et al. 2017, Ahmad et al. 2021, Murphy et al. 2021). More broadly, the organic carbon deposited from soil, faeces, saliva, and/or plant material in termite mounds likely drives microbial organoheterotrophic activity and growth (Holt 1998, Ndiaye et al. 2004a, Chiri et al. 2021). For example, the use of saliva as the cement medium in mound constructions could facilitate initial colonization, given it contains readily utilizable carbon (Gillman et al. 1972, Li et al. 2017).

Termite colonies also actively suppress invasion of fungal and bacterial pathogens in their nesting structures. Their faeces and saliva have antifungal properties, likely due to the production of antimicrobial compounds from both glandular secretions and microbiota activity within termites (Rosengaus et al. 2000, 2004). Their faeces also stimulates the growth of nest-associated antimicrobial-producing bacteria, e.g. *Streptomyces* (Chouvenc et al. 2013). Termites also rely on social immunity to limit infection: they use mutual grooming to reduce pathogen load; abandon and seal infected chambers in their compartmented nests; and identify, kill, cannibalize, or bury infected nestmates (Rosengaus et al. 1998, Traniello et al. 2002, Yanagawa and Shimizu 2007, Chouvenc et al. 2010, 2011, Chouvenc and Su 2012, Cremer et al. 2018). Altogether, this combination of activities allows termites to selectively exclude pathogens while favouring mutualists.

## Ecosystem services from termite-associated communities

In addition to providing multifaceted benefits to termites, the microbial communities associated with termite nesting structures offer a range of ecosystem services (Fig. 1). Of these, the most established is the regulation of greenhouse gas emissions. Hydrogenotrophic methanogens within the termite gut account for approximately 3% global methane emissions (Zimmerman et al. 1982, Rasmussen and Khalil 1983, Seiler et al. 1984, Brauman et al. 1992, Sugimoto et al. 1998a, Kirschke et al. 2013). While methane oxidation is negligible within the termite gut, approximately half of all termite-derived methane is oxidized by mound bacteria, thereby significantly reducing methane emissions (Fig. 3; Sugimoto et al.1998b, Pester et al. 2007, Ho et al. 2013, Nauer et al.2018b, Chiri et al. 2020, 2021). This process is primarily mediated by soil-recruited *Methylocapsa* species (USCα) and *Methylocystis* methanotrophs, which appear to be kinetically adapted to elevated methane concentrations (Chiri et al. 2020). In contrast to the numerous mound-associated hydrogenotrophs, deterministic factors appear to limit the abundance of methanotrophs, and hence they only partially mitigate emissions (Chiri et al. 2020, 2021). Mound-associated bacteria are also predicted to mediate hydrogenotrophic carbon dioxide fixation and reduce nitrous oxide (Chiri et al. 2021). However, further studies are required to measure whether these activities occur and whether they mitigate the substantial termite-derived emissions of these gases (Khalil et al. 1990, Jamali et al. 2011)

Termite-associated bacteria also enhance soil fertility, especially in savanna and dryland regions, by mediating various supporting services. Termite nest and mound soils promote growth of a wide range of plants both in natural environments and agricultural settings (Watson 1977, Arshad 1982, Rajagopal 1983, Mokossesse et al. 2012). It was traditionally thought that enhanced fertility is due to the increased nutrients present in nesting structures primarily as a result of termite decomposition (Batalha et al. 1995, López-Hernández 2001, Adhikary et al. 2016). However, increasing evidence suggests growth-promoting microbial communities also play a key role (Enagbonma and Babalola 2020). Plants make mutualistic associations with mound-associated microorganisms, most notably ectomycorrhizal fungi, and also likely benefit from enhanced nutrient acquisition (including nitrogen fixation and phosphate solubilization), regulation of metal availability, and disease regulation (via antimicrobial compounds) provided by the wider community (Spain et al. 2004, Duponnois et al. 2005, 2006a,2006b, Fox-Dobbs et al. 2010, Chakdar et al. 2018, Devi and Thakur 2018, Enagbonma and Babalola 2019a). The long-term turnover of nest and mound soils, e.g. through termite activity or erosion, naturally fertilizes surrounding soils (Wood 1988, De Bruyn and Conacher 1990). Some smallholders in Africa and Asia already apply termite mound soils to increase agricultural production, providing a relatively affordable and sustainable fertilization mechanism (Miyagawa et al. 2011, Menichetti et al. 2014, Apori et al. 2020). Mound soils can also be used to enhance rates of composting and bioremediation (Karak et al. 2014, Enagbonma and Babalola 2019a). These concepts are explored in more detail in several recent reviews (Enagbonma and Babalola 2019a, 2019b, Subi and Sheela 2020).

With respect to provisioning services, termite nesting communities are also relevant for understanding and tackling the antimicrobials arm race. Hundreds of novel natural products have been identified from termite nests, especially combs of fungus-farming termites, often with potent antibacterial or antifungal properties (Sujada et al. 2014, Schmidt et al. 2022). This finding suggests termite-associated bacteria are a valuable resource for novel antimicrobial discovery with a range of ‘one health’ applications. Antibiotic resistance genes are also found in termite mound communities, likely driven by bacterial and fungal antagonism. However, despite high levels of natural products and biosynthetic gene clusters observed, levels of antibiotic resistance genes are reportedly lower in mounds compared to surrounding soils (Yan et al. 2021). As ancient and successful exploiters of antibiotics, deeper studies are required to understand how insects maintain effective antimicrobial defences amid pathogen diversification and antimicrobial resistance.

## Summary

Increasing evidence suggests that the microbial communities residing within termite nesting structures engage in symbiotic relationships with termites. The microorganisms benefit from a relatively stable and selective habitat, high nutrient availability, and pathogen exclusion mechanisms. The microorganisms recruited to termite nesting structures likely span the spectrum of commensals to mutualists, though the majority appear to benefit termites by mediating antimicrobial production, lignocellulose digestion, and nutrient recycling. Yet in other respects, mound-associated bacteria are atypical of classical symbionts: they are generalist bacteria that can adopt both free-living and mound-associated lifestyles; are opportunistically recruited and sustained through normal nest-building and digestive activities of termites; and exhibit strong geographic variations in community composition even within species. However, strong arguments have been presented that animals can dependably acquire microbial symbionts if potential symbionts are widely distributed and promiscuous associations can form (Moran and Sloan 2015, Chouvenc et al. 2018). Likewise, mutualism need not depend on pairwise interactions and can instead involve interacting guilds (Stanton 2003). The termite-engineered microbial communities in these nesting structures also have broader environmental influences, both locally (e.g. through enhancing soil fertility) and globally (e.g. through mitigating greenhouse gas emissions), providing a further dimension of the concept of the ‘extended phenotype’ (Dawkins 2016).

## Notes

Invited review for FEMS Microbiology Reviews.
